# Efficacy and toxicity profile of first‐line treatment for extensive‐stage small cell lung cancer: A Bayesian network meta‐analysis

**DOI:** 10.1002/cam4.5750

**Published:** 2023-03-23

**Authors:** Guo Lin, Zhuoran Yao, Kai Kang, Hui Wang, Ren Luo, You Lu

**Affiliations:** ^1^ Thoracic Oncology Ward, Cancer Center, West China Hospital Sichuan University Chengdu, Sichuan China; ^2^ Laboratory of Clinical Cell Therapy, West China Hospital Sichuan University Chengdu, Sichuan China

**Keywords:** extensive‐stage small cell lung cancer, first‐line, immunotherapy, network meta‐analysis

## Abstract

**Background:**

The efficacy and toxicity profiles for extensive‐stage small cell lung cancer (ES‐SCLC) are unclear. We aimed to address this gap through a Bayesian network meta‐analysis.

**Methods:**

We performed network analysis from randomized controlled trials comparing these treatments: PD‐(L)1 inhibitor, CTLA‐4 inhibitor, CXCR inhibitor, PARP inhibitor, CDK inhibitor, chemotherapy, and their combinations. Pooled estimations of progression‐free survival, overall survival, objective response rate, and toxicity (systematic and specific) were conducted within the Bayesian framework.

**Results:**

Twenty‐five trials involving 9 strategies were included. In terms of progression‐free survival and overall survival, PD‐(L)1 inhibitor combined with cisplatin/carboplatin (P) and etoposide (E) shown the acknowledged superiority than other treatments. The addition of CTLA‐4 inhibitor (ipilimumab) to EP had the highest response rate among these regimens, and the combination of chemotherapy (irinotecan) and cisplatin/carboplatin had the greatest probability of performing considerable systematic security. The secondary endpoint was specific adverse events, including vomiting, fatigue, thrombocytopenia, constipation, and decreased appetite; hence we depicted the specific toxicity profile of each regimen. In addition, we identified the differences between PD‐1 inhibitors and PD‐L1 inhibitors in prolonging overall survival time for the central nervous system (CNS)/liver metastases patients.

**Conclusions:**

EP combined with PD‐(L)1 inhibitor followed by CTLA‐4 inhibitors or anti‐angiogenesis was the considerable treatment with considerable efficacy and safety for ES‐SCLC. Each treatment has a unique specific toxicity profile, which needs more attention.

## Graphical abstract

We compared 9 regimens and depicted the toicity profiles for treatment‐naive extensive stage small cell lung cancer patients.

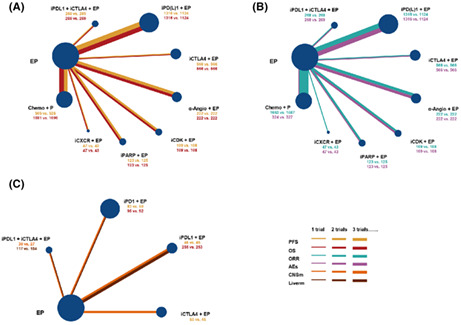

## 1 INTRODUCTION

Lung cancer remains a severe disease with high mortality and morbidity worldwide.^1^ Small cell lung cancer (SCLC) accounts for approximately 15% of lung cancer, with rapid progression and early metastasis, which is the most aggressive subtype of lung cancer.^2^ Generally, SCLC was divided into two subtypes: limited‐stage SCLC (LS‐SCLC) and extensive‐stage SCLC (ES‐SCLC).^3^ More than two‐thirds of patients were at the extensive stage at the time of diagnosis and carried a high concentration of circulating tumor cells in peripheral blood, and the 5‐year survival rate is <5%.^4^ Over the past decades, the combination of cisplatin/carboplatin and etoposide (EP) is the standard treatment option.^5^ In the beginning, SCLC had a considerable response (about 70%), but most ES‐SCLC patients relapse and/or metastasize within 1 year.^6^ Therefore, ES‐SCLC was regarded as a therapeutically challenging disease and imperatively needed a breakthrough in treatment. More combined immunotherapy modes are worth exploring, including the combination of immunotherapy, tyrosine kinase inhibitors, radiotherapy, anti‐vascular therapy. And the applications of cell‐free DNA methylation and single‐cell sequencing contribute to precise screenings of benefit populations.

In recent years, immune checkpoint inhibitors (ICIs) made remarkable progress in improving the prognosis of lung cancer.^7^, ^8^ Since IMpower133^9^ and CISPIAN^10^ trials reported the inspiring breakthroughs of first‐line ES‐SCLC treatment, emerging research demonstrated the significant benefits of combined therapies, including the addition of anti‐programmed cell death 1 (PD‐1), anti‐programmed cell death ligand 1 (PD‐L1), anti‐cytotoxic T‐cell lymphocyte antigen 4 (CTLA‐4), and anti‐vascular endothelial growth factor (VEGF) antibody.^11^ And the ASTRUM‐005 trial reported that serplulimab added to EP significantly improved survival time, decreased the incidence of disease progression and risk of death. Furthermore, some biological process inhibitors combined with EP also showed significant improvement in survival time compared to EP,^11^, ^12^ including cyclin‐dependent kinases (CDK) inhibitors and polyadenosine diphosphate ribose polymerase (PARP) inhibitors. PARP inhibitors can suppress DNA damage, enhance the activity of DNA‐targeting cancer‐restrain, and also shown favorable effects. Previous trials revealed survival benefits are accompanied by severs adverse events, even death.^13^, ^14^ Due to the short treatment window, choosing the optimal therapy and understanding the toxicity profiles are crucial for advanced patients.^15^

However, previous network meta‐analyses have compared part regimens for ES‐SCLC patients or used only direct comparison models,^16^ there was no study systematically depicting the efficacy and safety in first‐line treatment. Thus, we updated the survival time data and involve more regimens to perform this Bayesian network meta‐analysis, which can fully compare multiple therapeutic regimens by synthesizing direct or indirect comparison, attempting describe the prognostic profiles of all first‐line treatments in patients with ES‐SCLC.

## 2 MATERIALS AND METHODS

### 2.1 Literature search

According to the PRISMA (preferred reporting items for systematic reviews and meta‐analyses) guidelines and extension statement, we performed a systematic search through PubMed, Web of Science, Embase, and Cochrane Central Register of Controlled Trials databases to identify randomized controlled trials (RCTs) before 1 November 2022, there was no language limitations. The detailed search strategies were shown in Table S1. We also inspected meeting abstracts from World Conference on Lung Cancer, American Society of Clinical Oncology, and European Cancer Conference to update relevant data. The protocol was registered in the Prospective Register of Systematic Reviews (PROSPERO 394567).

### 2.2 Study selection

We collected published or unpublished studies which met the following criteria: (I) treatment‐naïve; (II) RCTs; (III) age 18 years or older; (IV) trials compared the efficacy or safety of multiple therapies based on EP; (V) patients with histologically or clinically documented ES‐SCLC (according to the eighth edition of American Joint Committee of Cancer staging guidelines); (VI) studies involved survival data as endpoints; Exclusion criteria: (I) uncompleted data; (II) case‐reports, letters, meta‐analyses and systematic reviews; (III) endpoint data could not be extracted or converted from the original research. Any discrepancies were resolved by consensus by a panel of adjudicators (G Lin, Z Yao, K Kang, H Wang, and R Luo).

### 2.3 Data extraction

Two researchers (G Lin, H Wang) independently screened information from eligible studies and recorded the following data into electronic spreadsheets: author, publication year, gender, age, smoke status, metastasis status, Eastern Cooperative Oncology Group (ECOG) score, efficacy endpoints included progression‐free survival (PFS), overall survival (OS), objective response rate (ORR), and toxicities were more than grade 3 treatment‐related adverse events (AEs) and all grade‐specific adverse events (s‐AEs).

### 2.4 Risk of bias assessment

The risk of bias assessment of each eligible trial was conducted by Review Manager (version 5.3) using Cochrane risk of bias tools, which considered these aspects: random sequence generation, allocation concealment, blinding of participants and personnel, blinding of outcome assessment, incomplete outcome data, selective reporting, and other bias. Items were categorized as low, high, or unclear risk of bias. Disagreements were solved by discussing with other researchers.

### 2.5 Statistical analysis

All direct and indirect data were collected to compare the efficacies and toxicities of each treatment. PFS and OS were the primary endpoints; ORR, AEs, and s‐AEs were the secondary outcomes. Hazard ratio for PFS and OS, and odds ratios (OR) for ORR and AEs. HR value <1 indicates a longer survival time, and OR value more than 1 suggests a lower incidence rate.

According to the sample size and the amount of study, network plots were produced by STATA (version SE 15.0) to illustrate the geometries. We generated forest plots for the direct comparison to estimate the pooled odds ratio and 95% confidence interval. The heterogeneity was assessed by inconsistency statistic (*I* ^2^) using the random‐effect model and *I* ^2^ more than 50% was considered high. Funnel plots were finished to observe publication bias.

Network meta‐analyses of efficacy and toxicity were conducted within the Bayesian consistency framework^17^ using Markov chain Monte Carlo method by OpenBUGs (version 3.2.3). Considering the significant heterogeneity among studies, the random‐effects model was applied to run the Bayesian analyses. Non‐informative uniform and normal prior distributions were set to fit the model, generating 100,000 iterations after a burn‐in of 50,000 in each chain of three independent chains (thinning interval = 10). The tools also estimate the overall rankings of each therapy by calculating the surface under the cumulative ranking curve area (SUCRA), the value indicates the probability of each regimen being the best, second best, or worst option.

## 3 RESULTS

### 3.1 Eligible researches and characteristics

Literature search identified 1253 relevant records from online databases and international conferences (Figure S1). After checking for titles/abstracts, a total of 25^9^, ^10^, ^18^, ^19^, ^20^, ^21^, ^22^, ^23^, ^24^, ^25^, ^26^, ^27^, ^28^, ^29^, ^30^, ^31^, ^32^, ^33^, ^34^, ^35^, ^36^, ^37^, ^38^, ^39^, ^40^, ^41^ randomized controlled trials met the criteria with a total of 8547 patients involved in receive 9 treatments including etoposide plus platin/carboplatin (EP), PD‐L1 (durvalumab) plus CTLA‐4 (tremelimumab) plus EP, PD‐(L)1 (pembrolizumab, durvalumab, atezolizumab, serplulimab, adebrelimab) plus EP, CTLA‐4 (ipilimumab) plus EP, anti‐angiogenesis (bevacizumab, rh‐Endostatin) plus EP, CDK inhibitor (trilaciclib, roniciclib) plus EP, PARP inhibitor (veliparib) plus EP, CXCR inhibitor (LY2510924) plus EP, and chemotherapeutics (amrubicin, topotecan, irinotecan) plus cisplatin/carboplatin. 17/26 studies were phase III clinical trials. All trials were double‐arm, except for the CASPIAN study (triple‐arm): EP, durvalumab plus EP, durvalumab plus tremelimumab plus EP. Totally, the network analyses included 9 treatment strategies for PFS, OS, ORR, and AEs/specific AEs. Eligible studies reported various specific AEs, and we are concerned 14 clinically relevant and data‐supported specific AEs, including the non‐hematologic events: vomiting, fatigue, constipation, decreased appetite, alopecia, nausea, dyspnea, diarrhea, and the hematologic parameters: white blood cell count decreased, anemia, leukopenia, thrombocytopenia, neutropenia. Detailed characteristics of eligible researches were summarized in Table 1. And see Figure S2 for detailed results of the bias assessment.

**TABLE 1:** Baseline characteristics of included studies in this network meta‐analysis.

Study	Sample size (n)	Phase	Median age (y)	CNS met (n)	Liver met (n)	Treatment		Type
Jonathan W. Goldman, 2021	268/269	3	63/63	38/27	117/104	Durvalumab plus tremelimumab plus platinum‐etoposide	Platinum‐etoposide	iPDL1 + iCTLA4 + EP versus EP
	268/269	3	62/63	28/27	108/104	Durvalumab plus platinum‐etoposide	Platinum‐etoposide	iPDL1 + EP versus EP
Charles M. Rudin, 2020	228/225	3	64/65	33/22	95/92	Pembrolizumab plus platinum‐etoposide	Platinum‐etoposide	iPD1 + EP versus EP
Stephen V. Liu, 2021	201/202	3	64/64	17/18	NR	Atezolizumab plus platinum‐etoposide	Platinum‐etoposide	iPDL1 + EP versus EP
Ying Cheng, 2022	389/196	3	63/62	50/28	99/51	Serplulimab plus platinum‐etoposide	Platinum‐etoposide	iPD1 + EP versus EP
Jie Wang, 2022	230/232	3	62/62	5/5	73/74	Adebrelimab plus platinum‐etoposide	Platinum‐etoposide	iPDL1 + EP versus EP
Martin Reck, 2016	478/476	3	62/63	55/45	NR	Ipilimumab plus platinum‐etoposide	Platinum‐etoposide	iCTLA4 + EP versus EP
David R. Spigel, 2011	52/50	2	64/60	2/1	NR	Bevacizumab plus platinum‐etoposide	Platinum‐etoposide	α‐Angiogenesis + EP versus EP
Marcello Tiseo, 2017	101/103	3	63/64	NR	NR	Bevacizumab plus platinum‐etoposide	Platinum‐etoposide	α‐Angiogenesis + EP versus EP
Shun Lu, 2015	69/69	2	57.7/58.2	NR	NR	Rh‐endostatin plus platinum‐etoposide	Platinum‐etoposide	α‐Angiogenesis + EP versus EP
J. M. Weiss, 2019	38/37	2	65/65	5/8	NR	Trilaciclib plus platinum‐etoposide	Platinum‐etoposide	iCDK + EP versus EP
Martin Reck, 2019	71/71	2	62/63	NR	NR	Roniciclib plus platinum‐etoposide	Platinum‐etoposide	iCDK + EP versus EP
Lauren Averett Byers, 2021	59/61	2	64/63	NR	NR	Veliparib plus platinum‐etoposide	Platinum‐etoposide	iPARP + EP versus EP
Taofeek Owonikoko, 2019	64/64	2	66/64	0/0	NR	Veliparib plus platinum‐etoposide	Platinum‐etoposide	iPARP + EP versus EP
Ravi Salgia, 2017	47/43	2	64/67	NR	NR	LY2510924 plus platinum‐etoposide	Platinum‐etoposide	iCXCR + EP versus EP
Yan Sun, 2016	149/150	3	58/59	30/17	37/41	Amrubicin plus platinum	Platinum‐etoposide	Chemo + P versus EP
Thomas H. Fink, 2012	346/334	3	61/61	NR	NR	Topotecan plus platinum	Platinum‐etoposide	Chemo + P versus EP
A. Schmittel, 2011	106/110	3	60/63	31/23	36/48	Irinotecan plus platinum	Platinum‐etoposide	Chemo + P versus EP
P. Zatloukal, 2010	202/203	3	59/60	30/22	82/90	Irinotecan plus platinum	Platinum‐etoposide	Chemo + P versus EP
Dong‐Wan Kim, 2019	173/189	3	66/65	70/75	NR	Irinotecan plus platinum	Platinum‐etoposide	Chemo + P versus EP
Yuank Shi, 2015	30/32	2	59/57	2/3	6/5	Irinotecan plus platinum	Platinum‐etoposide	Chemo + P versus EP
Primo N. Lara Jr, 2009	324/327	3	62/63	NR	NR	Irinotecan plus platinum	Platinum‐etoposide	Chemo + P versus EP
Andreas Hermes, 2008	105/104	3	67/68	17/12	46/48	Irinotecan plus platinum	Platinum‐etoposide	Chemo + P versus EP
Nasser Hanna, 2006	221/110	3	63/62	20/17	51/53	Irinotecan plus platinum	Platinum‐etoposide	Chemo + P versus EP
A. Schmittel, 2006	35/35	2	59/63	NR	NR	Irinotecan plus platinum	Platinum‐etoposide	Chemo + P versus EP
Kazumasa Noda, 2002	77/77	3	63/63	10/17	14/13	Irinotecan plus platinum	Platinum‐etoposide	Chemo + P versus EP

### 3.2 Network meta‐analysis in ES‐SCLC

**FIGURE 1:**
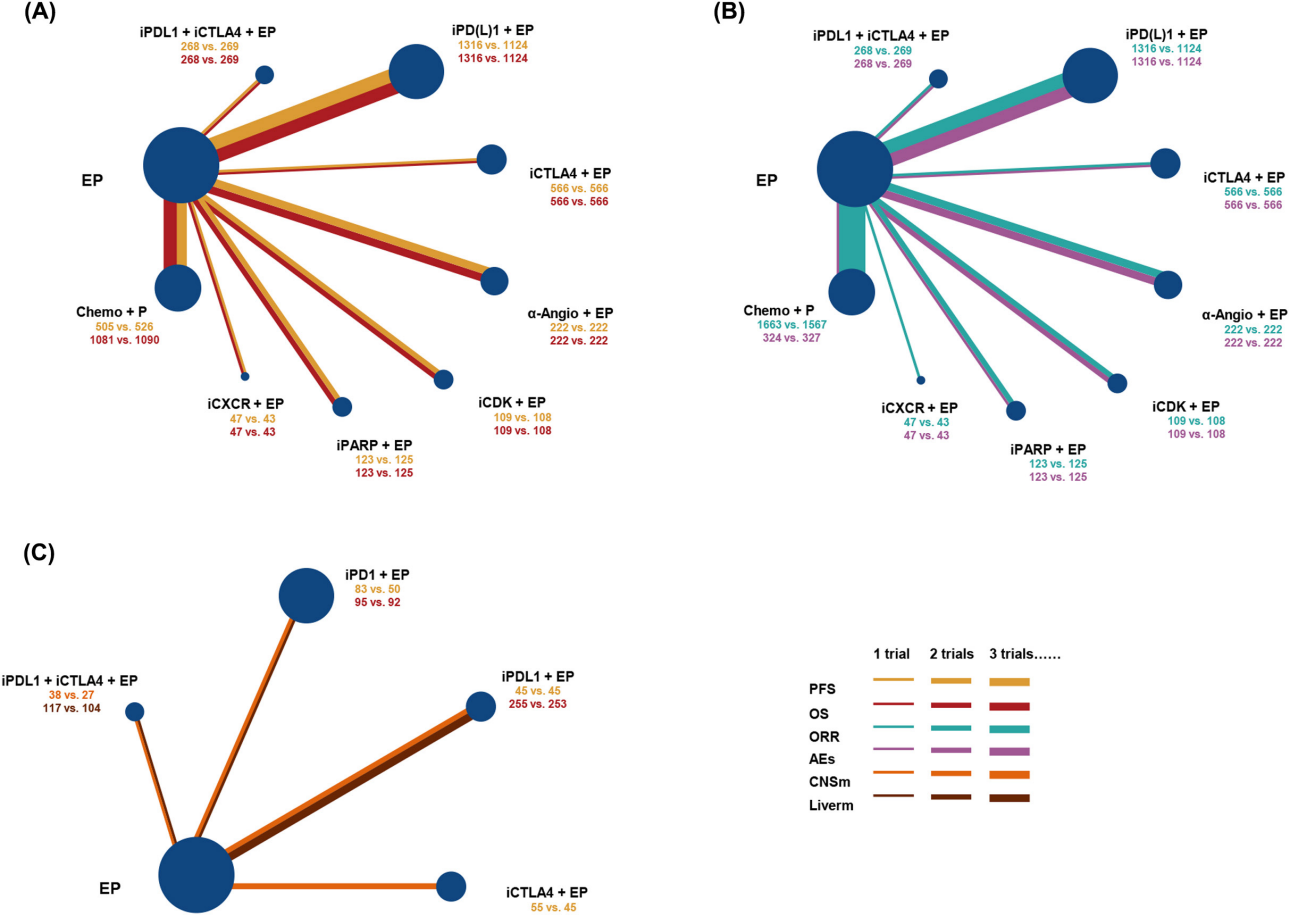
Network plot of included studies in this network meta‐analysis. Node size is proportional to the number of included patients (A, B) for overall patients and (C) for central nervous system and liver metastases patients. The width of lines indicates the number of trials, and color of line presents the endpoint (EP = cisplatin/carboplatin and etoposide, iPDL1 + iCTLA4 + EP = PDL1 inhibitor + CTLA4 inhibitor + EP, iPD(L)1 + EP = PD1/PDL1 inhibitor + EP, iCTLA4 + EP = CTLA4 inhibitor + EP, α‐Angio + EP = anti‐angiogenesis + EP, iCDK + EP = CDK inhibitor + EP, iPARP + EP = PARP inhibitor + EP, iCXCR + EP = CXCR inhibitor + EP, Chemo + P = Chemotherapy + P).

**FIGURE 2:**
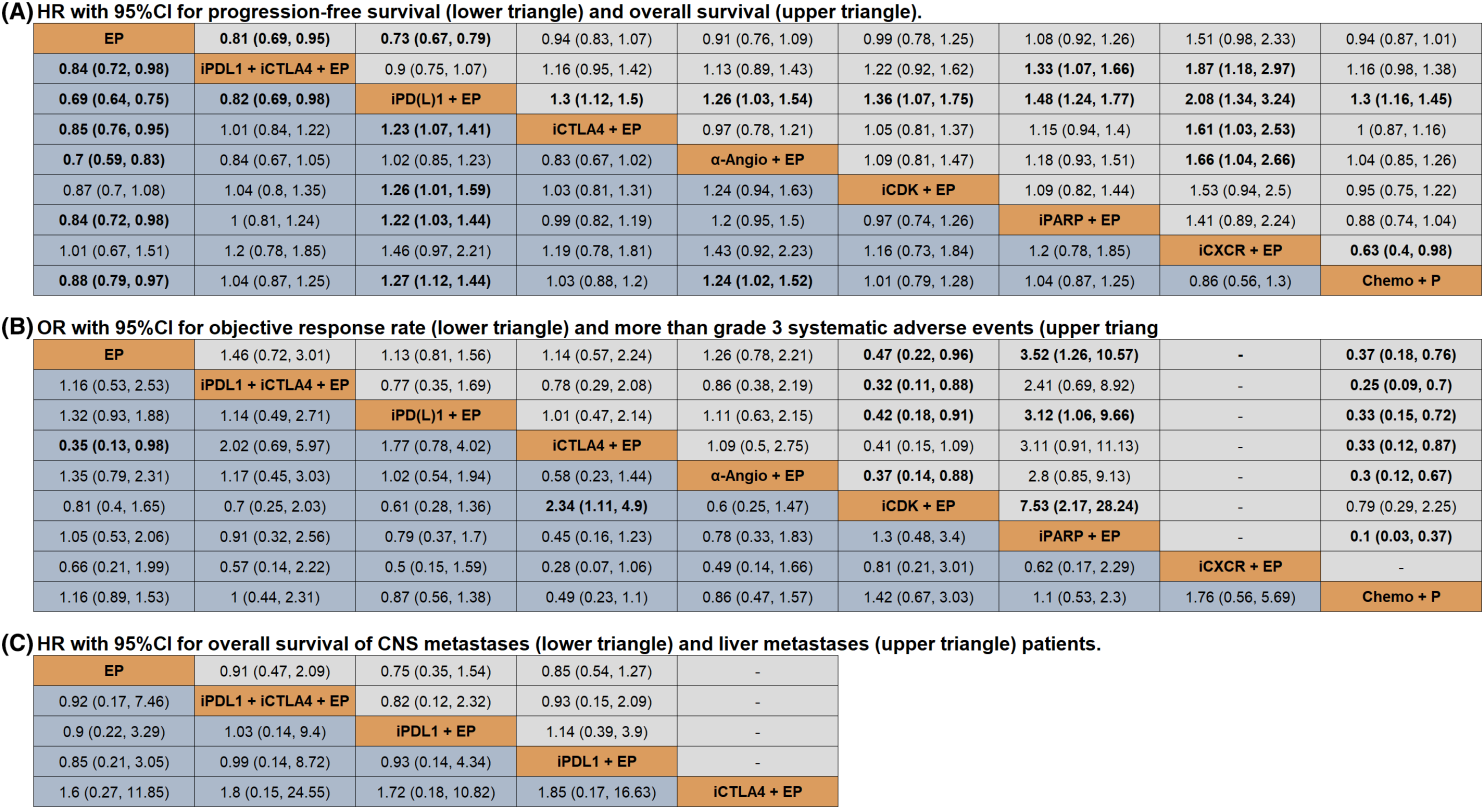
Pooled estimate results of (A) PFS and OS, (B) ORR and systematic AEs, (C) OS for metastases patients (EP = cisplatin/carboplatin and etoposide, iPDL1 + iCTLA4 + EP = PDL1 inhibitor + CTLA4 inhibitor + EP, iPD(L)1 + EP = PD1/PDL1 inhibitor + EP, iCTLA4 + EP = CTLA4 inhibitor + EP, α‐Angio + EP = anti‐angiogenesis + EP, iCDK + EP = CDK inhibitor + EP, iPARP + EP = PARP inhibitor + EP, iCXCR + EP = CXCR inhibitor + EP, Chemo + P = Chemotherapy + P).

All strategies involved in network meta‐analysis for PFS, OS, and ORR, and 8 treatments for more than grade 3 AEs (Figure 1A,B). According to *I* ^2^ value of heterogeneity test (Figure S3), we used a random‐effect model to perform the pooled estimate. In terms of PFS (Figure 2A), overall treatments are prior to EP, except for CDK inhibitor plus EP (HR = 0.87, 95% CI: 0.70–1.08) and CXCR inhibitor plus EP (HR = 1.01, 95% CI: 0.67–1.51). The combination of PD‐L1 and CTLA‐4 plus EP had a better PFS than PD‐(L)1 plus EP (HR = 0.82, 95% CI: 0.69–0.98). Additionally, CTLA‐4 plus EP (HR = 1.23, 95% CI: 1.07–1.41), CDK inhibitor plus EP (HR = 1.26, 95% CI: 1.01–1.59), PARP inhibitor plus EP (HR = 1.22, 95% CI: 1.03–1.44) and chemotherapy plus cisplatin/carboplatin (HR = 1.27, 95% CI: 1.12–1.44) performed the worse PFS than PD‐(L)1 plus EP. Anti‐angiogenesis drugs plus EP are significantly superior in prolonging PFS than chemotherapy plus cisplatin/carboplatin (HR = 1.24, 95% CI: 1.02–1.52). No remarkable difference was noticed among the combination of CTLA‐4, anti‐angiogenesis, CDK inhibitor, PARP inhibitor, and CXCR inhibitor with EP.

In terms of OS (Figure 2A), PD‐(L)1 significantly increased survival time compared with other treatments, except for the combination of PD‐L1 plus CTLA‐4 and EP (HR = 0.90, 95% CI: 0.75–1.07). And OS in PD‐L1 plus CTLA‐4 plus EP was significantly higher than that in EP (HR = 0.81, 95% CI: 0.69–0.95) and PARP inhibitor (HR = 1.33, 95% CI: 1.07–1.66). EP added to PD‐L1 plus CTLA‐4, PD‐(L)1, CTLA‐4, anti‐angiogenesis, and chemotherapy plus cisplatin/carboplatin were better than EP for prolonging OS.

In terms of ORR (Figure 2B), there was no typical discrepancy among treatments, except for CTLA‐4 plus EP shown a higher response rate than EP (OR = 0.35, 95% CI: 0.13–0.98) and CDK inhibitor plus EP (OR = 2.34, 95% CI: 1.11–4.90).

Regarding systematic AEs (Figure 2B), chemotherapy plus cisplatin/carboplatin had a lower toxicity rate than other treatments, apart from CDK inhibitor (OR = 0.79, 95% CI: 0.29–2.25). Besides, the AEs of CDK inhibitor plus EP are higher than other EP‐based therapies and equal to CTLA‐4 plus EP (OR = 0.41, 95% CI: 0.15–1.09). PARP inhibitor failed to decrease AEs rate compared with EP (OR = 3.52, 95% CI: 1.26–10.57) and PD‐(L)1 (OR = 3.12, 95% CI: 1.06–9.66). No significant difference was observed among EP and EP plus PD‐L1 plus CTLA‐4, PD‐(L)1, CTLA‐4, and anti‐angiogenesis.

### 3.3 Rank probabilities

**FIGURE 3:**
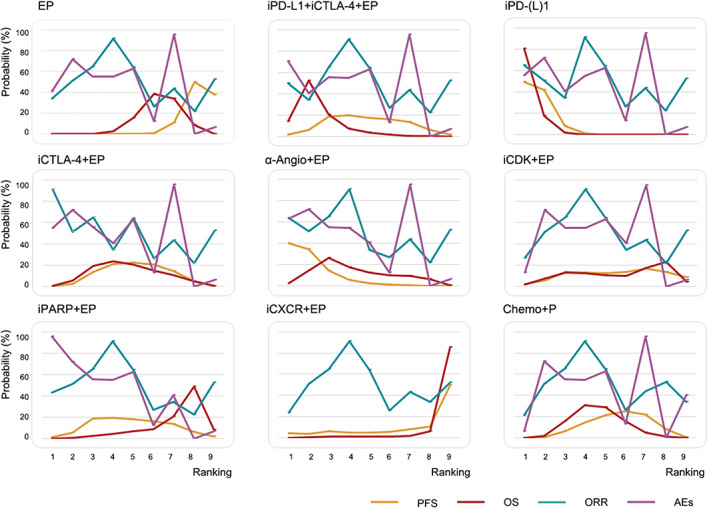
Ranking profiles of treatments according to original network meta‐analysis for overall patients (EP = cisplatin/carboplatin and etoposide, iPDL1 + iCTLA4 + EP = PDL1 inhibitor + CTLA4 inhibitor + EP, iPD(L)1 + EP = PD1/PDL1 inhibitor + EP, iCTLA4 + EP = CTLA4 inhibitor + EP, α‐Angio + EP = anti‐angiogenesis + EP, iCDK + EP = CDK inhibitor + EP, iPARP + EP = PARP inhibitor + EP, iCXCR + EP = CXCR inhibitor + EP, Chemo + P = Chemotherapy + P).

Figure 3 shows the Bayesian rank probabilities of all comparable therapies, suggesting efficacy and safety. The ranking outcomes were almost consistent with pooled analyses of PFS, OS, ORR, and AEs. PD‐(L)1 had the highest probability of improving PFS (cumulative probability 49.3%), and EP had the most significant probability of ranking last (cumulative probability 38.0%). Similar to PFS, PD‐(L)1 was related to the highest probability of ranking first for OS (cumulative probability 80.8%). CXCR inhibitor plus EP was most likely to be the worst regimen for improving OS. Bayesian ranking profiles indicated the combination of CTLA‐4 and EP was most possibly to be ranked first to offer the best ORR (cumulative probability 91.0%), followed by PD‐(L)1 plus EP (cumulative probability 64.9%).

In terms of safety, due to the lack of systematic AEs data of CXCR inhibitor plus EP, we presented the comparison among these 8 treatments. Chemotherapy plus cisplatin/carboplatin had the greatest probability of performing considerable security (cumulative probability 40.3%), and the combination of PARP inhibitor plus EP was most likely to lead to AEs (cumulative probability 95.4%).

### 3.4 Specific AEs

The outcomes of s‐AEs (all‐grade) analyses (Table 2) indicated that the addition of CTLA‐4 to PD‐L1 plus EP was similar to PD‐L1 plus EP. EP was safer than CTLA‐4 plus EP and anti‐angiogenesis plus EP for nausea and constipation, respectively. And EP was safer than chemotherapy plus cisplatin/carboplatin for fatigue, decreased appetite and diarrhea, but more toxic for thrombocytopenia and neutropenia. Furthermore, the combination of PD‐(L)1 and CTLA‐4 and EP had a higher constipation incidence rate than anti‐angiogenesis plus EP, and higher nausea rate than CDK inhibitor plus EP, higher diarrhea rate than chemotherapy combined with cisplatin/carboplatin. The combined therapy of CDK inhibitor had less fatigue and nausea, and more constipation and neutropenia than anti‐angiogenesis plus EP.

**TABLE 2:** Pooled estimate of specific adverse events (numbers in cells are odds ratios, <1 suggests the treatment is safer).

		Vomiting	Fatigue	Thrombocytopenia	Constipation	Decreased appetite	Alopecia	Nausea	Neutropenia	Anemia	Dyspnea	Diarrhea	Leukopenia
		6651	5687	6642	3004	5442	5543	7666	7021	8037	3390	6820	4780
iPDL1 + iCTLA4 + EP	versus EP	0.79	1.22	1.03	1.05	1.3	0.81	0.95	0.87	0.68	0.85	1.56	1.04
iPD(L)1 + EP		1.04	1.07	1.01	0.94	1.27	0.96	0.91	1.07	0.96	1.09	1.03	1.26
iCTLA4 + EP		1.5	1.19	0.95	NR	1.54	0.7	1.56*	0.67	0.78	NR	3.09	NR
α‐Angio + EP		0.96	0.53	0.71	4.76*	1.57	1.61	0.83	1.12	0.95	0.91	4.12	0.97
iCDK + EP		6.45*	1.79	0.96	0.5*	1.47	0.81	2.06*	0.07*	0.81	3.03*	3.95	NR
iPARP + EP		1.19	1.34	3.37	1.12	0.95	0.97	1.43	2.36	2.03	1.39	1.04	2.66
iCXCR + EP		NR	NR	NR	NR	NR	NR	NR	NR	0.88	NR	NR	NR
Chemo + P		0.91	1.49*	0.53*	1.38	1.79*	0.57	1.44	0.46*	0.8	1.09	7.12*	0.88
iPD(L)1 + EP	versus iPDL1 + iCTLA4 + EP	1.31	0.88	0.99	0.93	0.98	1.19	0.95	1.22	1.42	1.29	0.66	1.21
iCTLA4 + EP		1.91	0.97	0.93	NR	1.19	0.86	1.64	0.76	1.15	NR	1.98	NR
α‐Angio + EP		1.22	0.44	0.7	4.89*	1.2	1.99	0.87	1.28	1.4	1.08	2.70	0.93
iCDK + EP		8.2	1.46	0.94	0.47	1.12	1	2.17*	0.08	1.2	3.57	2.52	NR
iPARP + EP		1.52	1.1	3.27	1.03	0.73	1.2	1.51	2.7	2.99	1.63	0.67	2.56
iCXCR + EP		NR	NR	NR	NR	NR	NR	NR	NR	1.3	NR	NR	NR
Chemo + P		1.15	1.22	0.51	1.34	1.38	0.71	1.51	0.53	1.18	1.29	4.56*	0.85
iCTLA4 + EP	versus iPD(L)1 + EP	1.45	1.12	0.94	NR	1.22	0.72	1.72*	0.62	0.81	NR	2.99	NR
α‐Angio + EP		0.92	0.5	0.7	5.08*	1.23	1.67	0.91	1.05	0.99	0.83	4.04	0.77
iCDK + EP		6.22	1.68	0.95	0.52	1.15	0.84	2.27*	0.06*	0.84	2.77	3.82	NR
iPARP + EP		1.15	1.25	3.33	1.22	0.75	1.01	1.58*	2.22	2.1	1.26	1.01	2.11
iCXCR + EP		NR	NR	NR	NR	NR	NR	NR	NR	0.92	NR	NR	NR
Chemo + P		0.87	1.39	0.52	1.42	1.41	0.59	1.58	0.43*	0.83	1	6.90*	0.7
α‐Angio + EP	versus iCTLA4 + EP	0.64	0.45	0.75	NR	1.01	2.32	0.53	1.68	1.21	NR	1.36	NR
iCDK + EP		4.28	1.5	1.01	NR	0.95	1.17	1.32	0.1*	1.04	NR	1.28	NR
iPARP + EP		0.79	1.12	3.54	NR	0.61	1.39	0.91	3.53	2.6	NR	0.34	NR
iCXCR + EP		NR	NR	NR	NR	NR	NR	NR	NR	1.13	NR	NR	NR
iChemo + P		0.6	1.25	0.55	NR	1.16	0.82	0.92	0.69	1.03	NR	2.31	NR
iCDK + EP	versus α‐Angio + EP	6.72	3.38*	1.36	0.1*	0.94	0.5	2.47*	0.06*	0.86	3.35	0.93	NR
iPARP + EP		1.24	2.53	4.74	0.26	0.61	0.6	1.71	2.11	2.13	1.51	0.24	2.75
iCXCR + EP		NR	NR	NR	NR	NR	NR	NR	NR	0.93	NR	NR	NR
Chemo + P		0.95	2.79*	0.74	0.27	1.15	0.35	1.73	0.41	0.84	1.18	1.73	0.91
iPARP + EP	versus iCDK + EP	0.19	0.75	3.49	2.39	0.65	1.19	0.69	35.71*	2.5	0.46	0.26	NR
iCXCR + EP		NR	NR	NR	NR	NR	NR	NR	NR	1.09	NR	NR	NR
Chemo + P		0.14*	0.83	0.54	2.59	1.23	0.71	0.7	6.98	0.98	0.36	1.81	NR
iCXCR + EP	versus iPARP + EP	NR	NR	NR	NR	NR	NR	NR	NR	0.44	NR	NR	NR
Chemo + P		0.76	1.12	0.16	1.21	1.89	0.59	1.01	0.2	0.39	0.79	6.87*	0.33
Chemo + P	versus iCXCR + EP	NR	NR	NR	NR	NR	NR	NR	NR	0.91	NR	NR	NR

The significant values were bloded and asterisked.

Ranking profiles of s‐AEs (Table 3) suggested that the addition of PD‐L1 and CTLA‐4 to EP had the lowest probability of vomiting (probability 35%), anemia (33%), neutrophil (11%), and dyspnea (29%). Notably, CDK inhibitor added to EP was regarded as the safest regimen for constipation (6%) and neutropenia (1%). chemotherapy plus cisplatin/carboplatin was most likely to be the first to avoid thrombocytopenia (22%) and leukopenia (33%).

**TABLE 3:** Ranking profiles of each treatment in specific adverse events.

	Vomiting	Fatigue	Thrombocytopenia	Constipation	Decreased appetite	Alopecia	Nausea	Neutropenia	Anemia	Dyspnea	Diarrhea	Leukopenia
EP	0.43	0.29	0.54	0.44	0.17*	0.61	0.34	0.60	0.58	0.38	0.22*	0.41
iPDL1 + iCTLA4 + EP	0.35*	0.55	0.52	0.49	0.48	0.46	0.28	0.53	0.33*	0.29*	0.40	0.45
iPD(L)1 + EP	0.45	0.39	0.53	0.35	0.46	0.56	0.19*	0.63	0.53	0.47	0.25	0.56
iCTLA4 + EP	0.58	0.52	0.49	NA	0.64	0.39	0.76	0.43	0.40	NA	0.62	NA
α‐Angio + EP	0.41	0.07*	0.38	0.96	0.62	0.72	0.20	0.65	0.51	0.41	0.67	0.41
iCDK + EP	0.92	0.81	0.49	0.06*	0.57	0.47	0.88	0.01*	0.42	0.89	0.69	NA
iPARP + EP	0.49	0.65	0.83	0.52	0.26	0.55	0.64	0.91	0.88	0.59	0.27	0.84
iCXCR + EP	NA	NA	NA	NA	NA	NA	NA	NA	0.47	NA	NA	NA
Chemo + P	0.37	0.75	0.22*	0.68	0.80	0.24*	0.70	0.23	0.37	0.47	0.88	0.33*

The significant values were bloded and asterisked.

### 3.5 Subgroup analysis

**FIGURE 4:**
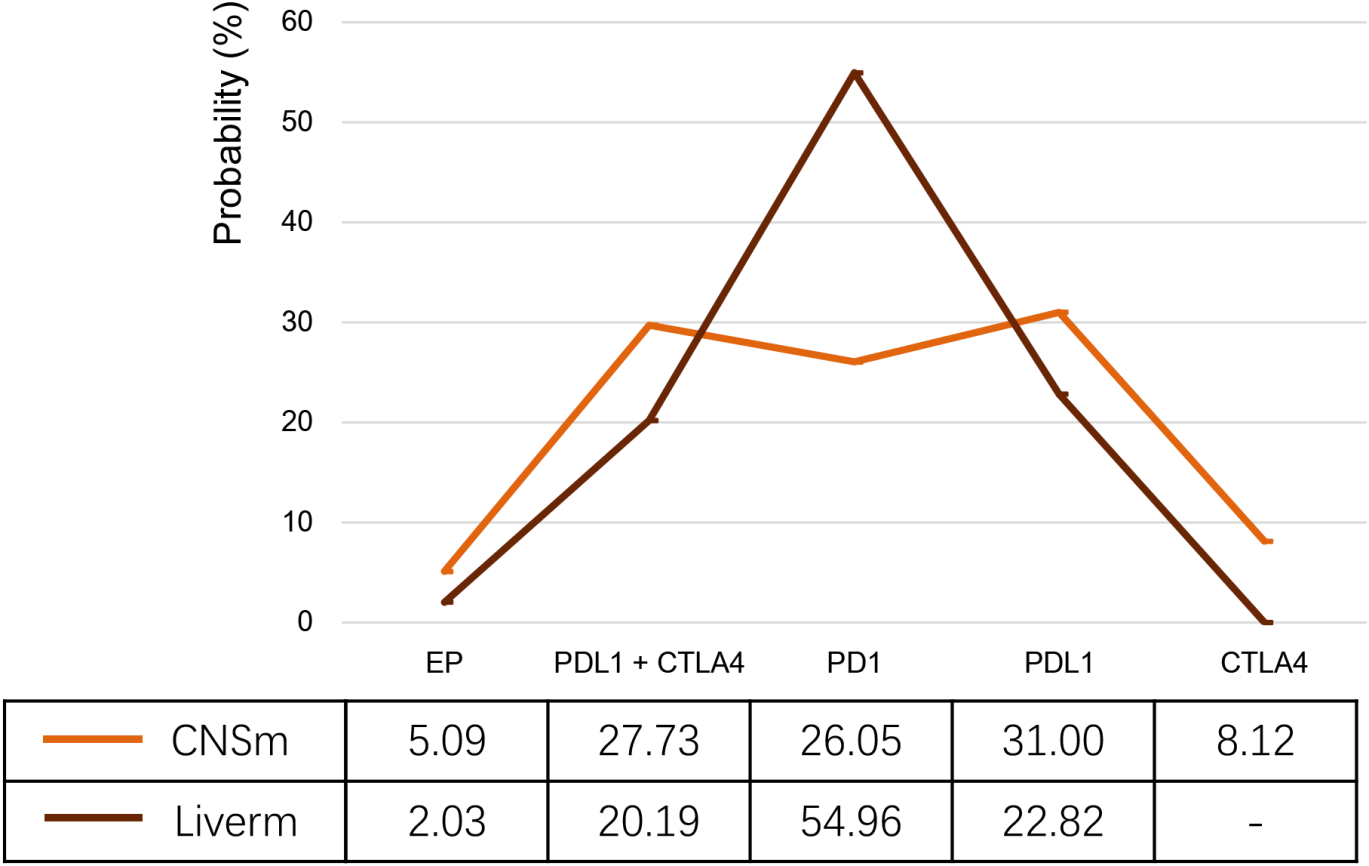
Ranking profiles of treatments according to subgroup network meta‐analysis for metastases patients (EP = cisplatin/carboplatin and etoposide, iPDL1 + iCTLA4 + EP = PDL1 inhibitor + CTLA4 inhibitor + EP, iPD1 + EP = PD1 inhibitor + EP, iPDL1 + EP = PDL1 inhibitor + EP, iCTLA4 + EP = CTLA4 inhibitor + EP).

Central nervous system (CNS) and liver metastases patients were involved in our subgroup analysis (Figure 1C). Limited by the published data, only five strategies (PD‐L1 inhibitor plus CTLA‐4 inhibitor plus EP, PD‐1 inhibitor plus EP, PD‐L1 inhibitor plus EP, CTLA‐4 inhibitor plus EP, and EP monotherapy) were compared in prolonging OS time. There was no discrepancy among them (Figure 2C). The Bayesian ranking profiles (Figure 4) indicated that PD‐L1 inhibitor plus EP had the most probability of being the best regimen in improving survival time for CNS metastases patients, and PD‐1 inhibitor (pembrolizumab) plus EP showed the greatest probability of ranking first in OS time comparison for liver metastases patients.

### 3.6 Heterogeneity and inconsistency assessment

Sensitive analyses were performed after excluded phase II trials. 6 treatments for 5344 untreated ES‐SCLC patients were included in analyses. The results were stable and were similar to the main analysis after excluding 9 trials. The outcomes of the consistency model were a better fit for our analyses (Table S2). Funnel plots demonstrated no obvious publication bias existed (Figure S4).

## 4 DISCUSSION

In this systematic review and network meta‐analysis, we updated data and comprehensively performed the comparison of multiple first‐line treatments, including PD‐(L)1, CTLA‐4, anti‐angiogenesis, CDK inhibitor, CXCR inhibitor, PARP inhibitor, chemotherapy, EP, and their combination regimens for treatment‐naïve ES‐SCLC. The results suggested that:

1. PD‐(L)1 inhibitors combined with EP were the optimal choice for prolonging survival time.
2. CTLA‐4 (ipilimumab) plus EP presented the highest response rate and was followed by PD‐(L)1 inhibitor regimens based on EP.
3. The combination of chemotherapy (irinotecan) and cisplatin showed less toxicity in general and also had considerable performance in specific adverse events.
4. PD‐L1 and PD‐1 inhibitors had the highest probability of improving OS for CNS and liver metastases patients, respectively.

The previous study reported no significant survival difference between PD‐1 inhibitors and PD‐L1 inhibitors between various regimens (pembrolizumab, durvalumab, atezolizumab) for overall ES‐SCLC patients in first‐line treatment^42^; therefore, we merged their data when conducting NMA. Similar to general acknowledgment, PD‐(L)1 inhibitors have shown distinguished superiority in increasing survival time.^43^ For the overall population or metastases patients, the combination therapy of PD‐L1 inhibitor and CTLA‐4 inhibitor failed to present an advantage over PD‐L1 inhibitor or CTLA‐4 inhibitor in terms of survival time or safety. The following reasons might explain the finding. Previous studies reported that CTLA‐4 mainly regulates T‐cell activation in lymph nodes and/or tissues and inhibits dendritic cell activity by regulatory T cells (Tregs).^44^ Meanwhile, PD‐1 is involved in inhibiting the activation of effector T cells and natural killer cells in peripheral tissues and inducing the differentiation of Tregs.^45^ Therefore, the addition of PD‐1 to CTLA‐4 has a synergistic effect on the activation of T cells and tumor‐restrain immune response. Also, US FDA approved the dual therapy of nivolumab and ipilimumab for the management of malignant cancers.^46^ However, it is important that PD‐1 inhibitors and PD‐L1 inhibitors had distinct mechanisms in anti‐tumor, such as the different express location.^47^, ^48^ And most of searches tested the combination of nivolumab and ipilimumab and the only minority of studies evaluated other anti‐PD‐1/PD‐L1 regimens combined with CTLA‐4 inhibitors,^49^, ^50^, ^51^ the credible mechanisms of durvalumab plus tremelimumab is still unclear. Consistent with existing studies, durvalumab combined with tremelimumab showed a higher incidence rate of systematic and specific AEs in this NMA,^46^ which may be alleviated by changing the administration sequence or dose of drugs.

In terms of CNS and liver metastases patients, we also demonstrated the slight distinction between PD‐1 and PD‐L1 inhibitors. The efficacy of immunotherapy depends on various tumor‐progression mechanisms and the complex tumor microenvironment^52^; then we considered the individual heterogeneity among metastases and patients. Moreover, we did not observe similarity among these immunotherapies (anti‐PD‐1, anti‐PD‐L1, and anti‐CTLA‐4), which was in accord with the preceding conclusion: the poor efficacy of immunotherapy for advanced patients. SCLC, as intractable cancer, has “cool” tumor features, is characterized by low immunogenicity, less CD8^+^ T cell infiltration and inhibitory tumor microenvironment, therefore, the inability of immunotherapy to reverse the anti‐tumor function of T cells.^53^ For advanced patients, dysfunction and/or insufficient quantity of effector T cells is the main factor of unfavorable therapeutic efficacy.^54^, ^55^ Recent studies demonstrated that the advantages of immunotherapy administration come from recruiting fresh T cells from adjacent lymph nodes to tumor microenvironment.^56^ Hence, attracting functional T cells to the tumor area is the key to improving therapeutic efficacy, which needs further exploration.

The analyses of systematic and specific AEs suggested the safety of irinotecan followed by CDK inhibitor (trilaciclib, roniciclib), and the greater toxicity of PARP inhibitor (veliparib). The detailed ranking also indicated that each strategy is related to different toxicity spectrums, which subsequent explanations, the differences in biological mechanisms of each treatment strategy might illustrate. Immune checkpoint inhibitors are different from anti‐angiogenesis drugs. Immunotherapy is more likely to induce immune‐related side effects, and chemotherapy is associated with alopecia and hypocytosis.^57^ Understanding the toxicity spectrums of first‐line treatments is crucial to clinical decisions, including combination therapies. Formulating a suitable treatment plan can avoid the accumulation of toxicities and even anesis or counteract. Physicians can choose the most suitable, individual treatment plan for patients, especially elders, based on their baseline conditions and intentions, is contributed to enhancing compliance.

Our research also has some limitations. First, all data were from indirect comparison and there was no drug closed loop; therefore, the assumption of transitivity is inevitable. External validation of our study is necessary. Second, limited to the published data, we are unable to consider confounding factors such as age, race, smoking status, and so on. The baseline characteristics are related to efficacy, but existing data are insufficient to conduct subgroup analysis. Third, some trials involved a small sample size, which certainly affects the robustness of results, in particular subgroup analysis for metastases patients. Lastly, some trials are continued, and published data cannot be included in the complete analysis and cannot represent the final results exactly. For example, the Impower133 trial just updated the OS and safety data, but PFS and ORR in 2021. To summarize, further studies are needed to support our continued research for ES‐SCLC.

## 5 CONCLUSION

This network meta‐analysis comprehensively compares various first‐line treatments and describes the toxicity figure. PD‐(L)1 inhibitor based on EP is the superior choice, followed by anti‐angiogenesis for most ES‐SCLC patients and in first‐line treatment. Dual immunotherapy fails to show superiority over monotherapy. CTLA‐4 inhibitor (ipilimumab) is associated with better response, and irinotecan has the safest profile among these strategies. We also depicted the specific AEs spectrum of each treatment. These findings provide the efficacy and safety reference for individualized treatment for ES‐SCLC patients.

## AUTHOR CONTRIBUTIONS

**Guo Lin:** Conceptualization (equal); data curation (equal); formal analysis (equal); investigation (equal); methodology (equal); software (equal); validation (equal); writing – original draft (equal). **Zhuoran Yao:** Data curation (equal); formal analysis (equal); methodology (equal); writing – original draft (equal). **Kai Kang:** Data curation (equal); investigation (equal); writing – original draft (equal). **Hui Wang:** Validation (equal); writing – original draft (equal). **Ren Luo:** Methodology (equal); supervision (equal); writing – original draft (equal). **You Lu:** Conceptualization (equal); project administration (equal); supervision (equal).

## FUNDING INFORMATION

This work was funded by 1·3·5 project for disciplines of excellence, West China Hospital, Sichuan University (ZYJC21003) and the National Natural Science Foundation of China (grants 82003242, 82073336) and Sichuan Provincial Research Foundation (No. 21YYJC2403).

## CONFLICT OF INTEREST STATEMENT

The authors declare that they have no conflicts of interest.

## Supporting information


Data S1

## Data Availability

Not applicable.
