# Supplementary material for: Efficacy and toxicity profile of first‐line treatment for extensive‐stage small cell lung cancer: A Bayesian network meta‐analysis
Source: Cancer Med. 2023 Mar 23;12(9):10230–42. doi: 10.1002/cam4.5750 (PMC10225202; doi:10.1002/cam4.5750)
Supplement: Supplementary file 1 — Data S1 [file CAM4-12-10230-s001.pdf]

Figure S1. Flowchart of this network meta-analysis.

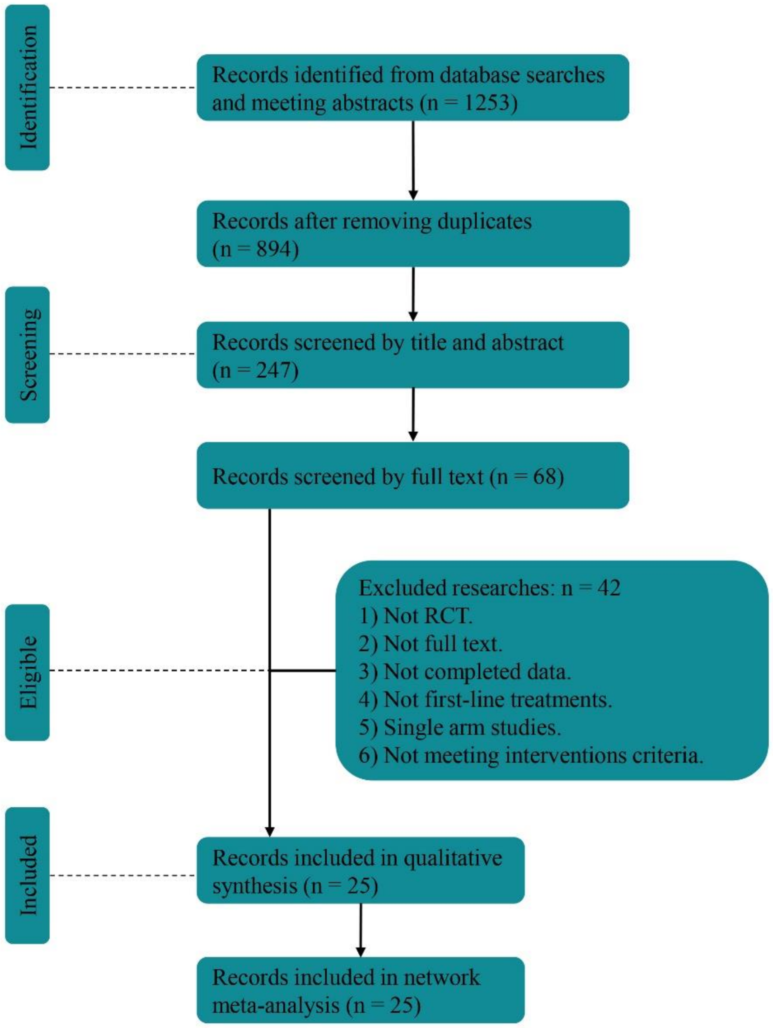

Figure S2. Risk of bias assessment

A. Risk of bias summary: judgements about each risk of bias item for each included study.

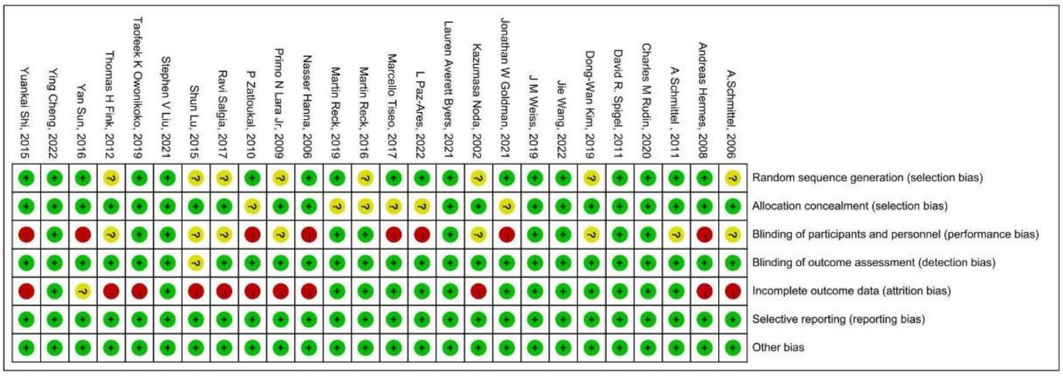

B. Risk of bias graph: judgements (low, unclear and high) about each risk of bias item presented as percentages across all included studies.

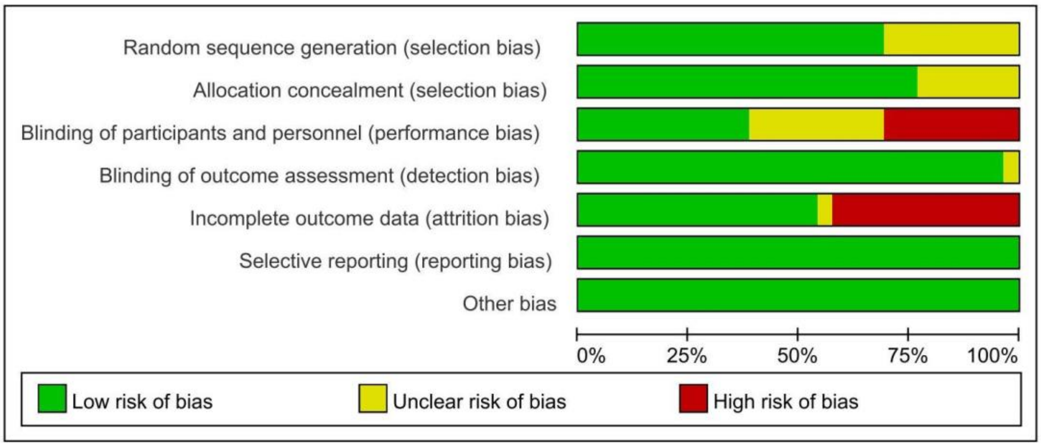

Figure S3. Forest plots for pairwise meta-analysis of head-to-head comparisons, (A) progression-free survival, (B) overall survival, (C) objective response rate, (D) more than grade 3 systematic adverse events.

(A)

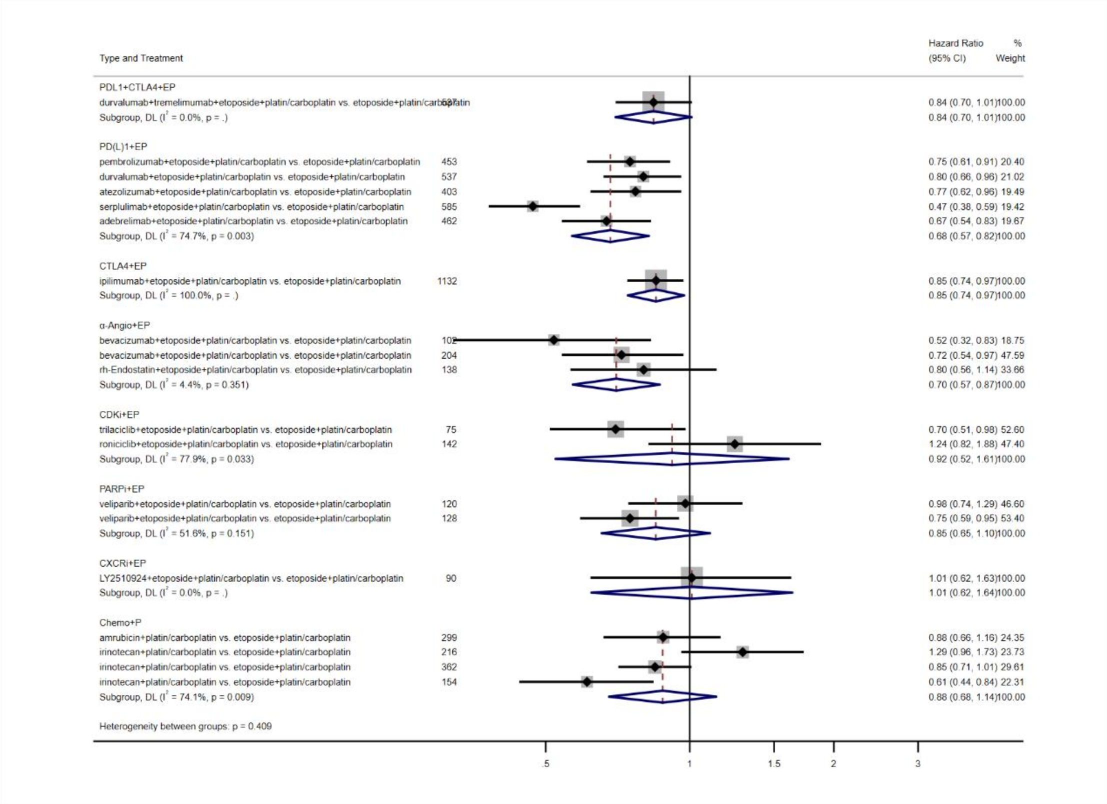

(B)

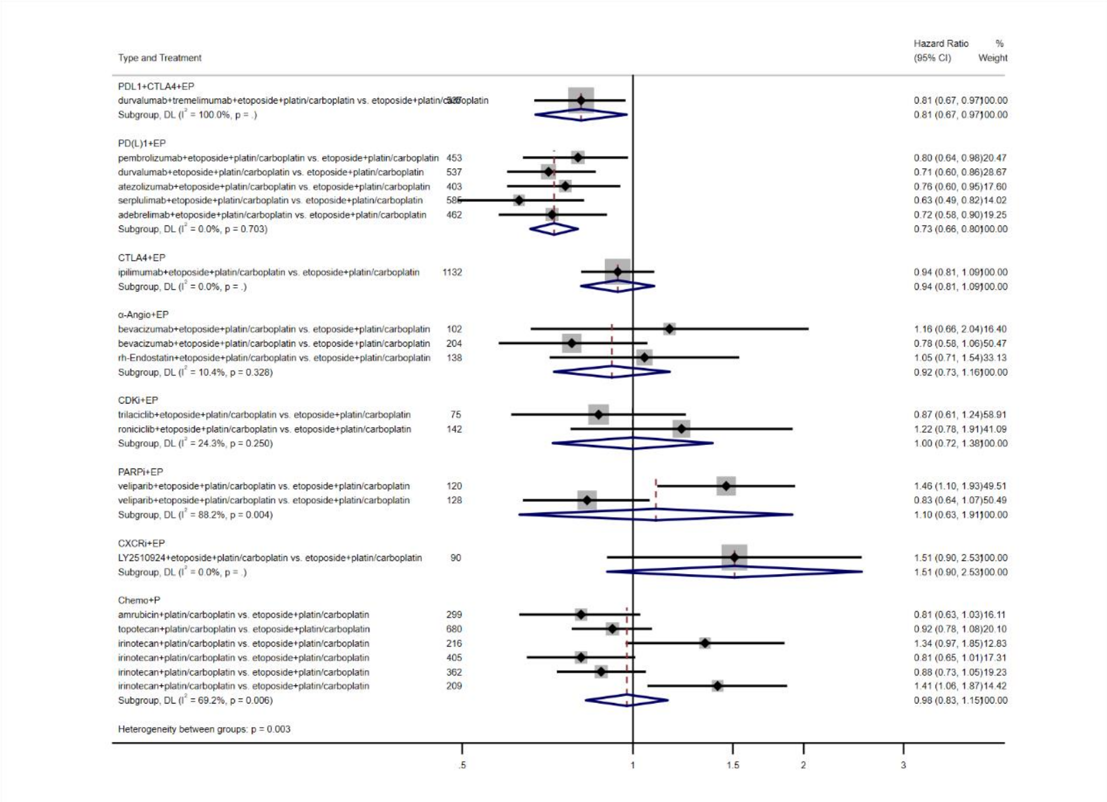

(C)

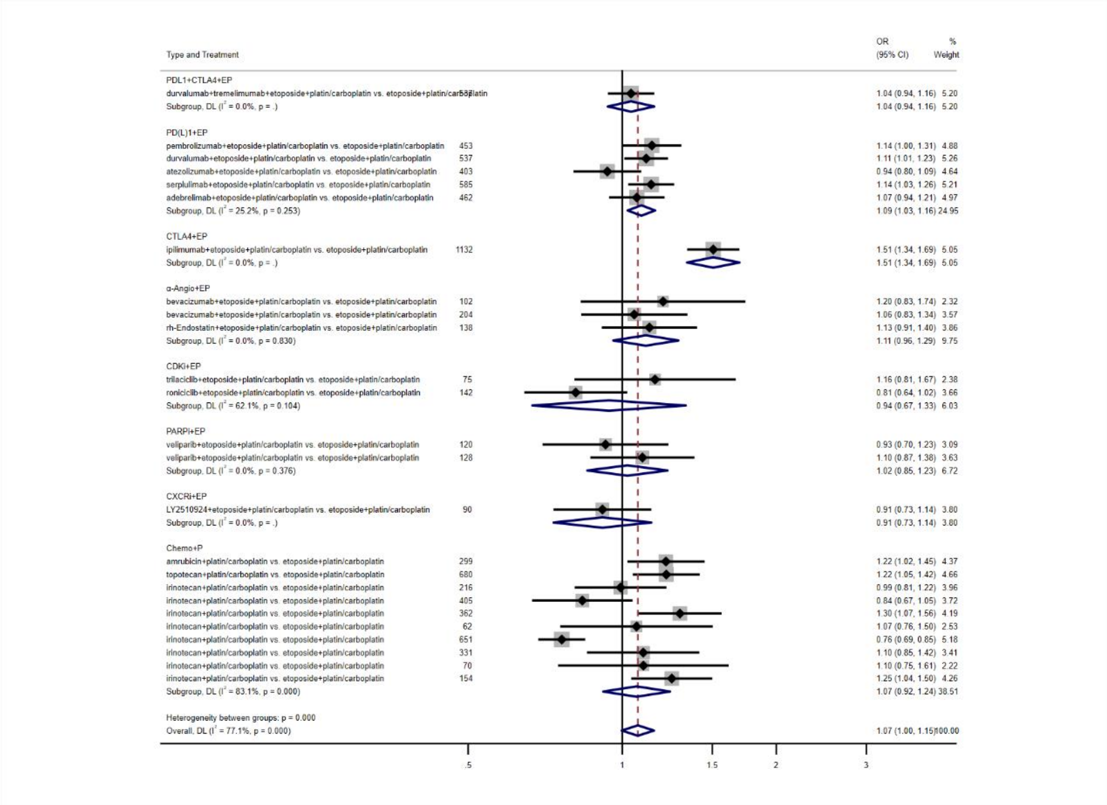
Type and Treatment
OR (95% CI)
% Weight
PDL1+CTLA4+EP
durvalumab+tremelimumab+etoposide+platin/carboplatin vs. etoposide+platin/carboplatin 537
1.04 (0.94, 1.16) 5.20
Subgroup, DL (I² = 0.0%, p = .)
1.04 (0.94, 1.16) 5.20
PD(L)1+EP
pembrolizumab+etoposide+platin/carboplatin vs. etoposide+platin/carboplatin 453
1.14 (1.00, 1.31) 4.88
durvalumab+etoposide+platin/carboplatin vs. etoposide+platin/carboplatin 537
1.11 (1.01, 1.23) 5.26
atezolizumab+etoposide+platin/carboplatin vs. etoposide+platin/carboplatin 403
0.94 (0.80, 1.09) 4.64
serplulimab+etoposide+platin/carboplatin vs. etoposide+platin/carboplatin 585
1.14 (1.03, 1.26) 5.21
adebrelimab+etoposide+platin/carboplatin vs. etoposide+platin/carboplatin 462
1.07 (0.94, 1.21) 4.97
Subgroup, DL (I² = 25.2%, p = 0.253)
1.09 (1.03, 1.16) 24.95
CTLA4+EP
ipilimumab+etoposide+platin/carboplatin vs. etoposide+platin/carboplatin 1132
1.51 (1.34, 1.69) 5.05
Subgroup, DL (I² = 0.0%, p = .)
1.51 (1.34, 1.69) 5.05
α-Angio+EP
bevacizumab+etoposide+platin/carboplatin vs. etoposide+platin/carboplatin 102
1.20 (0.83, 1.74) 2.32
bevacizumab+etoposide+platin/carboplatin vs. etoposide+platin/carboplatin 204
1.06 (0.83, 1.34) 3.57
rh-Endostatin+etoposide+platin/carboplatin vs. etoposide+platin/carboplatin 138
1.13 (0.91, 1.40) 3.86
Subgroup, DL (I² = 0.0%, p = 0.830)
1.11 (0.96, 1.29) 9.75
CDKi+EP
trilaciclib+etoposide+platin/carboplatin vs. etoposide+platin/carboplatin 75
1.16 (0.81, 1.67) 2.38
roniciclib+etoposide+platin/carboplatin vs. etoposide+platin/carboplatin 142
0.81 (0.64, 1.02) 3.66
Subgroup, DL (I² = 62.1%, p = 0.104)
0.94 (0.67, 1.33) 6.03
PARPi+EP
veliparib+etoposide+platin/carboplatin vs. etoposide+platin/carboplatin 120
0.93 (0.70, 1.23) 3.09
veliparib+etoposide+platin/carboplatin vs. etoposide+platin/carboplatin 128
1.10 (0.87, 1.38) 3.63
Subgroup, DL (I² = 0.0%, p = 0.376)
1.02 (0.85, 1.23) 6.72
CXCRi+EP
LY2510924+etoposide+platin/carboplatin vs. etoposide+platin/carboplatin 90
0.91 (0.73, 1.14) 3.80
Subgroup, DL (I² = 0.0%, p = .)
0.91 (0.73, 1.14) 3.80
Chemo+P
amrubicin+platin/carboplatin vs. etoposide+platin/carboplatin 299
1.22 (1.02, 1.45) 4.37
topotecan+platin/carboplatin vs. etoposide+platin/carboplatin 680
1.22 (1.05, 1.42) 4.66
irinotecan+platin/carboplatin vs. etoposide+platin/carboplatin 216
0.99 (0.81, 1.22) 3.96
irinotecan+platin/carboplatin vs. etoposide+platin/carboplatin 405
0.84 (0.67, 1.05) 3.72
irinotecan+platin/carboplatin vs. etoposide+platin/carboplatin 362
1.30 (1.07, 1.56) 4.19
irinotecan+platin/carboplatin vs. etoposide+platin/carboplatin 62
1.07 (0.76, 1.50) 2.53
irinotecan+platin/carboplatin vs. etoposide+platin/carboplatin 651
0.76 (0.69, 0.85) 5.18
irinotecan+platin/carboplatin vs. etoposide+platin/carboplatin 331
1.10 (0.85, 1.42) 3.41
irinotecan+platin/carboplatin vs. etoposide+platin/carboplatin 70
1.10 (0.75, 1.61) 2.22
irinotecan+platin/carboplatin vs. etoposide+platin/carboplatin 154
1.25 (1.04, 1.50) 4.26
Subgroup, DL (I² = 83.1%, p = 0.000)
1.07 (0.92, 1.24) 38.51
Heterogeneity between groups: p = 0.000
Overall, DL (I² = 77.1%, p = 0.000)
1.07 (1.00, 1.15) 100.00
.5
1
1.5
2
3

(D)

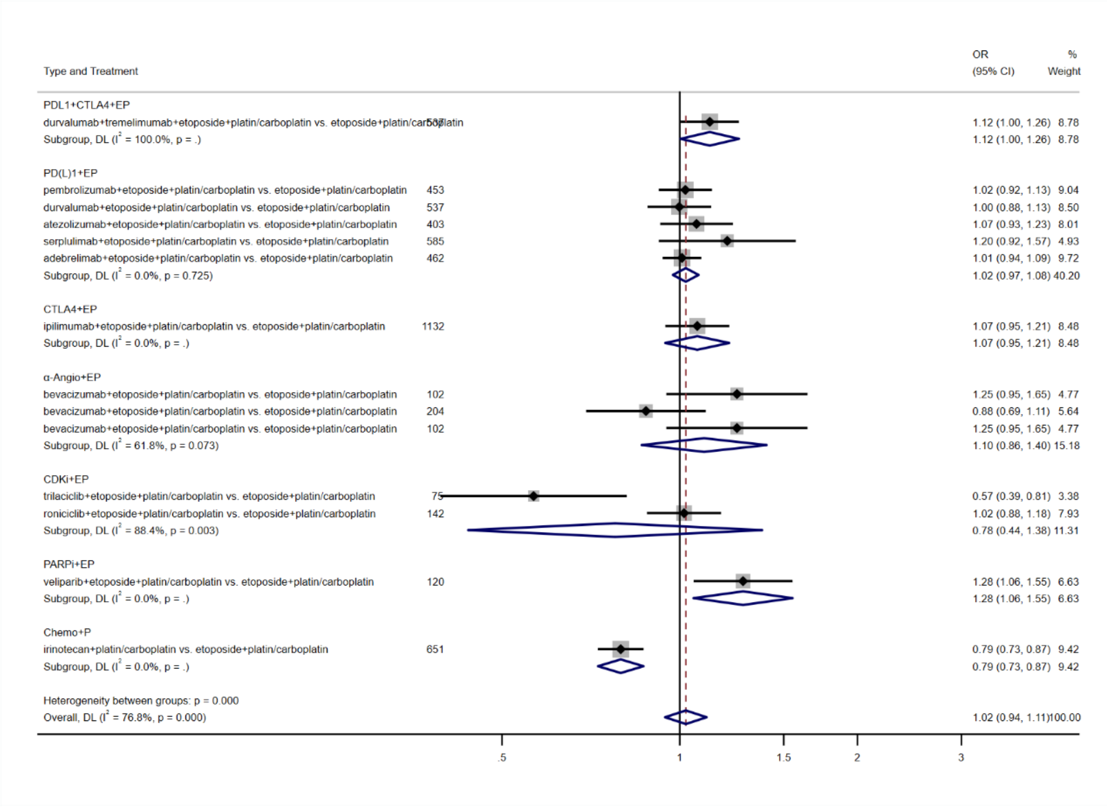
Type and Treatment
OR (95% CI)
% Weight
PDL1+CTLA4+EP
durvalumab+tremelimumab+etoposide+platin/carboplatin vs. etoposide+platin/carboplatin 537
1.12 (1.00, 1.26) 8.78
Subgroup, DL (I² = 100.0%, p = .)
1.12 (1.00, 1.26) 8.78
PD(L)1+EP
pembrolizumab+etoposide+platin/carboplatin vs. etoposide+platin/carboplatin 453
1.02 (0.92, 1.13) 9.04
durvalumab+etoposide+platin/carboplatin vs. etoposide+platin/carboplatin 537
1.00 (0.88, 1.13) 8.50
atezolizumab+etoposide+platin/carboplatin vs. etoposide+platin/carboplatin 403
1.07 (0.93, 1.23) 8.01
serplulimab+etoposide+platin/carboplatin vs. etoposide+platin/carboplatin 585
1.20 (0.92, 1.57) 4.93
adebrelimab+etoposide+platin/carboplatin vs. etoposide+platin/carboplatin 462
1.01 (0.94, 1.09) 9.72
Subgroup, DL (I² = 0.0%, p = 0.725)
1.02 (0.97, 1.08) 40.20
CTLA4+EP
ipilimumab+etoposide+platin/carboplatin vs. etoposide+platin/carboplatin 1132
1.07 (0.95, 1.21) 8.48
Subgroup, DL (I² = 0.0%, p = .)
1.07 (0.95, 1.21) 8.48
α-Angio+EP
bevacizumab+etoposide+platin/carboplatin vs. etoposide+platin/carboplatin 102
1.25 (0.95, 1.65) 4.77
bevacizumab+etoposide+platin/carboplatin vs. etoposide+platin/carboplatin 204
0.88 (0.69, 1.11) 5.64
bevacizumab+etoposide+platin/carboplatin vs. etoposide+platin/carboplatin 102
1.25 (0.95, 1.65) 4.77
Subgroup, DL (I² = 61.8%, p = 0.073)
1.10 (0.86, 1.40) 15.18
CDKi+EP
trilaciclib+etoposide+platin/carboplatin vs. etoposide+platin/carboplatin 75
0.57 (0.39, 0.81) 3.38
roniciclib+etoposide+platin/carboplatin vs. etoposide+platin/carboplatin 142
1.02 (0.88, 1.18) 7.93
Subgroup, DL (I² = 88.4%, p = 0.003)
0.78 (0.44, 1.38) 11.31
PARPi+EP
veliparib+etoposide+platin/carboplatin vs. etoposide+platin/carboplatin 120
1.28 (1.06, 1.55) 6.63
Subgroup, DL (I² = 0.0%, p = .)
1.28 (1.06, 1.55) 6.63
Chemo+P
irinotecan+platin/carboplatin vs. etoposide+platin/carboplatin 651
0.79 (0.73, 0.87) 9.42
Subgroup, DL (I² = 0.0%, p = .)
0.79 (0.73, 0.87) 9.42
Heterogeneity between groups: p = 0.000
Overall, DL (I² = 76.8%, p = 0.000)
1.02 (0.94, 1.11) 100.00
.5
1
1.5
2
3

Figure S4. Funnel plots of included trials in this network meta-analysis.

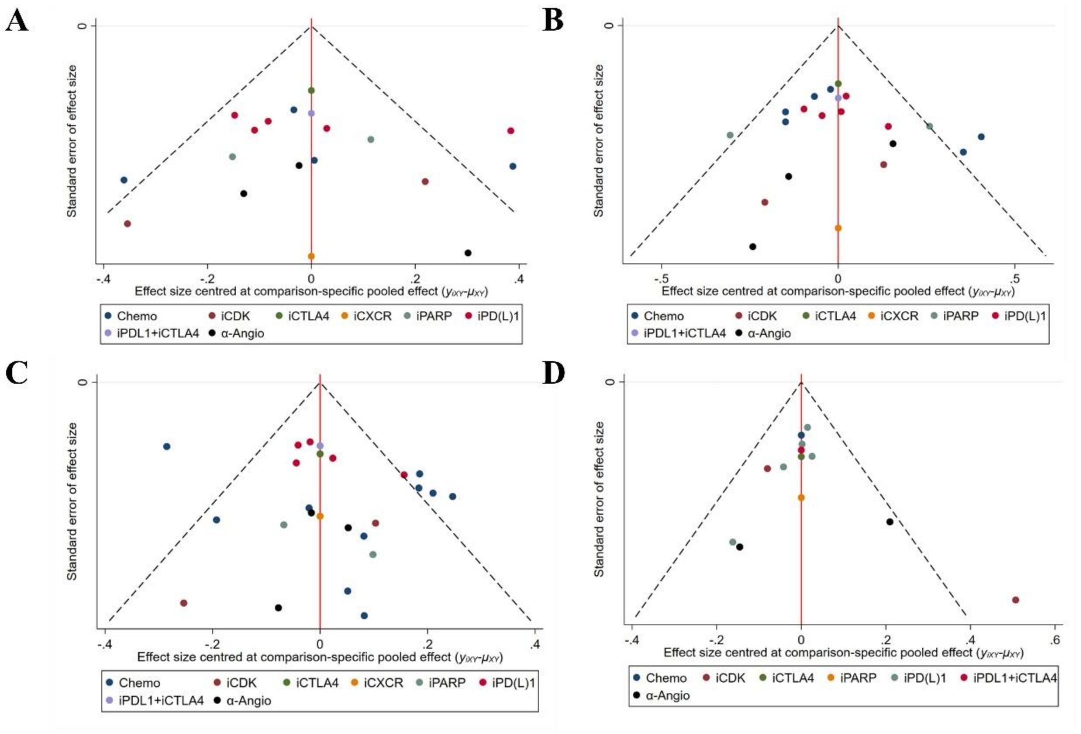

Table S1. Literature search strategy.

**PubMed**	
#1	"small cell lung cancer"[Title/Abstract] OR "extensive-stage small cell lung cancer"[Title/Abstract] OR "extensive-disease small cell lung cancer"[Title/Abstract]
#2	"immunotherapy"[Title/Abstract] OR "anti-programmed cell death 1"[Title/Abstract] OR "anti-programmed cell death ligand 1"[Title/Abstract] OR "anti-cytotoxic T-cell lymphocyte antigen 4"[Title/Abstract] OR "anti-vascular endothelial growth factor"[Title/Abstract] OR durvalumab[Title/Abstract] OR tremelimumab[Title/Abstract] OR pembrolizumab[Title/Abstract] OR atezolizumab[Title/Abstract] OR serplulimab[Title/Abstract] OR adebrelimab[Title/Abstract] OR ipilimumab[Title/Abstract] OR bevacizumab[Title/Abstract] OR "CDK inhibitor" [Title/Abstract] OR trilaciclib[Title/Abstract] OR roniciclib[Title/Abstract] OR veliparib[Title/Abstract] OR LY2510924[Title/Abstract] OR irinotecan[Title/Abstract] OR amrubicin[Title/Abstract] OR topotecan[Title/Abstract]
#3	"Randomized Controlled Trial"[Publication Type] OR "Clinical Trial"[Publication Type] OR trial[Title/Abstract] OR randomized[Title/Abstract] OR randomised[Title/Abstract] OR randomly[Title/Abstract] OR phase[Title/Abstract]
#4	"0001/01/01"[Date - Publication] : "2022/10/30"[Date - Publication]
#5	#1 AND #2 AND #3 AND #4
**Web of Science**	
#1	TS=("small cell lung cancer" OR "extensive-stage small cell lung cancer" OR "extensive-disease small cell lung cancer")<br>Databases= WOS, KJD, MEDLINE, RSCI, SCIELO Timespan=All years
#2	TS=("immunotherapy" OR "anti-programmed cell death 1" OR "anti- programmed cell death ligand 1" OR "anti-cytotoxic T-cell lymphocyte antigen 4" OR "anti-vascular endothelial growth factor" OR durvalumab OR tremelimumab OR pembrolizumab OR atezolizumab OR serplulimab OR adebrelimab OR ipilimumab OR bevacizumab OR "CDK inhibitor" OR trilaciclib OR roniciclib OR veliparib OR LY2510924 OR irinotecan OR amrubicin OR topotecan)<br>Databases= WOS, KJD, MEDLINE, RSCI, SCIELO Timespan=All years
#3	TS=(random* OR "clinical trial")<br>Databases= WOS, KJD, MEDLINE, RSCI, SCIELO Timespan=All years
#4	#1 AND #2 AND #3
**Embase**	
#1	'small cell lung cancer':ab,ti OR 'extensive-stage small cell lung cancer':ab,ti OR 'extensive-disease small cell lung cancer' :ab,ti
#2	'immunotherapy':ab,ti OR 'anti-programmed cell death 1':ab,ti OR 'anti- programmed cell death ligand 1':ab,ti OR 'anti-cytotoxic T-cell lymphocyte antigen 4':ab,ti OR 'anti-vascular endothelial growth factor':ab,ti OR 'durvalumab':ab,ti OR 'tremelimumab':ab,ti OR 'pembrolizumab':ab,ti OR 'atezolizumab':ab,ti OR 'serplulimab':ab,ti OR 'adebrelimab':ab,ti OR 'ipilimumab':ab,ti OR 'bevacizumab':ab,ti OR 'CDK inhibitor':ab,ti OR 'trilaciclib':ab,ti OR 'roniciclib':ab,ti OR 'veliparib':ab,ti OR 'LY2510924':ab,ti OR 'irinotecan':ab,ti OR 'amrubicin':ab,ti OR 'topotecan':ab,ti
#3	[controlled clinical trial]/lim OR [randomized controlled trial]/lim
#4	#1 AND #2 AND #3
**Cochrane Library**	
#1	"small cell lung cancer" OR "extensive-stage small cell lung cancer" OR "extensive-disease small cell lung cancer" in Title Abstract Keyword (Word variations have been searched)
#2	"immunotherapy" OR "anti-programmed cell death 1" OR "anti- programmed cell death ligand 1" OR "anti-cytotoxic T-cell lymphocyte antigen 4" OR "anti-vascular endothelial growth factor" OR durvalumab OR tremelimumab OR pembrolizumab OR atezolizumab OR serplulimab OR

	adebrelimab OR ipilimumab OR bevacizumab OR “CDK inhibitor” OR trilaciclib OR roniciclib OR veliparib OR LY2510924 OR irinotecan OR amrubicin OR topotecan in Title Abstract Keyword (Word variations have been searched)
#3	"randomized controlled trial" AND "randomized-controlled trial" in Publication Type - with Cochrane Library publication date to July 2020 (Word variations have been searched)
#4	#1 AND #2 AND #3
**ClinicalTrials.gov**	
#1	(“small cell lung cancer” OR “extensive-stage small cell lung cancer” OR “extensive-disease small cell lung cancer”) AND (“immunotherapy” OR “anti-programmed cell death 1” OR “anti- programmed cell death ligand 1” OR “anti-cytotoxic T-cell lymphocyte antigen 4” OR “anti-vascular endothelial growth factor” OR durvalumab OR tremelimumab OR pembrolizumab OR atezolizumab OR serplulimab OR adebrelimab OR ipilimumab OR bevacizumab OR “CDK inhibitor” OR trilaciclib OR roniciclib OR veliparib OR LY2510924 OR irinotecan OR amrubicin OR OSI- topoteca) AND (randomized OR randomized OR randomly OR random OR phase)

Table S2. Comparisons of the fit of consistency and inconsistency models using deviance information criteria.

**Outcome**	**Model**	**Deviance Information Criterion (DIC)**	**Accepted Model**
Progression-free survival	Consistency	36.413	Consistency
	Inconsistency	36.491	
Overall survival	Consistency	39.383	Consistency
	Inconsistency	39.344	
Objective response rate	Consistency	93.691	Consistency
	Inconsistency	93.754	
Systematic adverse events (more than grade 3)	Consistency	52.665	Consistency
	Inconsistency	52.974	

Table S3. Checklist of PRISMA statement for systematic review involving a network meta-analysis.

Section/Topic	Item #	Checklist Item	Reported on Page #
**TITLE**			
Title	1	Identify the report as a systematic review *incorporating a network meta-analysis (or related form of meta-analysis).*	**1**
**ABSTRACT**			
Structured summary	2	Provide a structured summary including, as applicable:<br>**Background:** main objectives<br>**Methods:** data sources; study eligibility criteria, participants, and interventions; study appraisal; and *synthesis methods, such as network meta-analysis.*<br>**Results:** number of studies and participants identified; summary estimates with corresponding confidence/credible intervals; *treatment rankings may also be discussed. Authors may choose to summarize pairwise comparisons against a chosen treatment included in their analyses for brevity.*<br>**Discussion/Conclusions:** limitations; conclusions and implications of findings.<br>**Other:** primary source of funding; systematic review registration number with registry name.	**2**
**INTRODUCTION**			
Rationale	3	Describe the rationale for the review in the context of what is already known*, including mention of why a network meta-analysis has been conducted.*	**3**
Objectives	4	Provide an explicit statement of questions being addressed, with reference to participants, interventions, comparisons, outcomes, and study design (PICOS).	**3**
**METHODS**			
Protocol and registration	5	Indicate whether a review protocol exists and if and where it can be accessed (e.g., Web address); and, if available, provide registration information, including registration number.	**4**
Eligibility criteria	6	Specify study characteristics (e.g., PICOS, length of follow-up) and report characteristics (e.g., years considered, language, publication status) used as criteria for eligibility, giving rationale. *Clearly describe eligible treatments included in the treatment network, and note whether any have been clustered or merged into the same node (with justification).*	**4**
Information sources	7	Describe all information sources (e.g., databases with dates of coverage, contact with study authors to identify additional studies) in the search and date last searched.	**4**
Search	8	Present full electronic search strategy for at least one database, including any limits used, such that it could be repeated.	**Supplementary Materials page 6-7**
Study selection	9	State the process for selecting studies (i.e., screening, eligibility, included in systematic review, and, if applicable, included in the meta-analysis).	**4**
Data collection process	10	Describe method of data extraction from reports (e.g., piloted forms, independently, in duplicate) and any processes for obtaining and confirming data from investigators.	**5**

Data items	11	List and define all variables for which data were sought (e.g., PICOS, funding sources) and any assumptions and simplifications made.	**5**
**Geometry of the network**	**S1**	Describe methods used to explore the geometry of the treatment network under study and potential biases related to it. This should include how the evidence base has been graphically summarized for presentation, and what characteristics were compiled and used to describe the evidence base to readers.	**6**
Risk of bias within individual studies	12	Describe methods used for assessing risk of bias of individual studies (including specification of whether this was done at the study or outcome level), and how this information is to be used in any data synthesis.	**5**
Summary measures	13	State the principal summary measures (e.g., risk ratio, difference in means). *Also describe the use of additional summary measures assessed, such as treatment rankings and surface under the cumulative ranking curve (SUCRA) values, as well as modified approaches used to present summary findings from meta-analyses.*	**6**
Planned methods of analysis	14	Describe the methods of handling data and combining results of studies for each network meta-analysis. This should include, but not be limited to:<br>• *Handling of multi-arm trials;*<br>• *Selection of variance structure;*<br>• *Selection of prior distributions in Bayesian analyses; and*<br>• *Assessment of model fit.*	**6**
**Assessment of Inconsistency**	**S2**	Describe the statistical methods used to evaluate the agreement of direct and indirect evidence in the treatment network(s) studied. Describe efforts taken to address its presence when found.	**6**
Risk of bias across studies	15	Specify any assessment of risk of bias that may affect the cumulative evidence (e.g., publication bias, selective reporting within studies).	**6**
Additional analyses	16	Describe methods of additional analyses if done, indicating which were pre-specified. This may include, but not be limited to, the following:<br>• Sensitivity or subgroup analyses;<br>• Meta-regression analyses;<br>• *Alternative formulations of the treatment network; and*<br>• *Use of alternative prior distributions for Bayesian analyses (if applicable).*	**6**
**RESULTS†**			
Study selection	17	Give numbers of studies screened, assessed for eligibility, and included in the review, with reasons for exclusions at each stage, ideally with a flow diagram.	**6-7; Supplementary Materials page 1**
**Presentation of network structure**	**S3**	Provide a network graph of the included studies to enable visualization of the geometry of the treatment network.	**Figure 1**
**Summary of network geometry**	**S4**	Provide a brief overview of characteristics of the treatment network. This may include commentary on the abundance of trials and randomized patients for the different interventions and pairwise comparisons in the network, gaps of evidence in the treatment network, and potential biases reflected by the network structure.	**7-8**
Study characteristics	18	For each study, present characteristics for which data were extracted (e.g., study size, PICOS, follow-up period) and provide the citations.	**7-8**
Risk of bias within	19	Present data on risk of bias of each study and, if available, any outcome	**Supplementary**

studies		level assessment.	**Materials page 2**
Results of individual studies	20	For all outcomes considered (benefits or harms), present, for each study: 1) simple summary data for each intervention group, and 2) effect estimates and confidence intervals. *Modified approaches may be needed to deal with information from larger networks.*	**8**
Synthesis of results	21	Present results of each meta-analysis done, including confidence/credible intervals. *In larger networks, authors may focus on comparisons versus a particular comparator (e.g. placebo or standard care), with full findings presented in an appendix. League tables and forest plots may be considered to summarize pairwise comparisons.* If additional summary measures were explored (such as treatment rankings), these should also be presented.	**8-9; Figure 2; Figure 3; Supplementary Materials page 3-5**
**Exploration for inconsistency**	**S5**	Describe results from investigations of inconsistency. This may include such information as measures of model fit to compare consistency and inconsistency models, *P* values from statistical tests, or summary of inconsistency estimates from different parts of the treatment network.	**10; Supplementary Materials page 8**
Risk of bias across studies	22	Present results of any assessment of risk of bias across studies for the evidence base being studied.	**10; Supplementary Materials page 2**
Results of additional analyses	23	Give results of additional analyses, if done (e.g., sensitivity or subgroup analyses, meta-regression analyses*, alternative network geometries studied, alternative choice of prior distributions for Bayesian analyses,* and so forth).	**9-10; Figure 4**
**DISCUSSION**			
Summary of evidence	24	Summarize the main findings, including the strength of evidence for each main outcome; consider their relevance to key groups (e.g., healthcare providers, users, and policy-makers).	**10**
Limitations	25	Discuss limitations at study and outcome level (e.g., risk of bias), and at review level (e.g., incomplete retrieval of identified research, reporting bias). *Comment on the validity of the assumptions, such as transitivity and consistency. Comment on any concerns regarding network geometry (e.g., avoidance of certain comparisons).*	**12-13**
Conclusions	26	Provide a general interpretation of the results in the context of other evidence, and implications for future research.	**13**
**FUNDING**			
Funding	27	Describe sources of funding for the systematic review and other support (e.g., supply of data); role of funders for the systematic review. This should also include information regarding whether funding has been received from manufacturers of treatments in the network and/or whether some of the authors are content experts with professional conflicts of interest that could affect use of treatments in the network.	**13**

PICOS = population, intervention, comparators, outcomes, study design.

*** Text in italics indicateS wording specific to reporting of network meta-analyses that has been added to guidance from the PRISMA statement.**

† Authors may wish to plan for use of appendices to present all relevant information in full detail for items in this section.
